# Sample Preparation and Diagnostic Methods for a Variety of Settings: A Comprehensive Review

**DOI:** 10.3390/molecules26185666

**Published:** 2021-09-18

**Authors:** Zach E. Nichols, Chris D. Geddes

**Affiliations:** 1 Department of Chemistry and Biochemistry, University of Maryland, Baltimore County, 1000 Hilltop Drive, Baltimore, MD 21250, USA; z36@umbc.edu; 2 Institute of Fluorescence, University of Maryland, Baltimore County, 701 E Pratt Street, Baltimore, MD 21270, USA

**Keywords:** sample preparation, point-of-care (POC), medical diagnosis, point-of-care testing (POCT), high-throughput screening, extraction, separations, microfluidics, biosensors

## Abstract

Sample preparation is an essential step for nearly every type of biochemical analysis in use today. Among the most important of these analyses is the diagnosis of diseases, since their treatment may rely greatly on time and, in the case of infectious diseases, containing their spread within a population to prevent outbreaks. To address this, many different methods have been developed for use in the wide variety of settings for which they are needed. In this work, we have reviewed the literature and report on a broad range of methods that have been developed in recent years and their applications to point-of-care (POC), high-throughput screening, and low-resource and traditional clinical settings for diagnosis, including some of those that were developed in response to the coronavirus disease 2019 (COVID-19) pandemic. In addition to covering alternative approaches and improvements to traditional sample preparation techniques such as extractions and separations, techniques that have been developed with focuses on integration with smart devices, laboratory automation, and biosensors are also discussed.

## 1. Introduction

Nearly every analytical assay in use today requires some type of sample pretreatment or preparation in order to transform samples from their collected form into a form suitable for analysis [1,2,3,4]. While the target analytes and underlying theories of analytical procedures vary greatly, most assays consist of sample collection, isolation of target analytes, detection of the targets, quantification, and the interpretation and handling of the resulting data [1,2,4,5,6,7]. In most cases, sample preparation is taken to mean any operations performed on a sample prior to instrumental analysis, typically consisting of the separation of target analytes from some matrices, the concentration of analytes, and the chemical or physical modifications made to improve downstream separation or detection [1,2,4]. It is worth noting that while there is no official agreement on the terms, sample preparation is generally associated with the chemical modifications to a sample while sample pretreatment is usually associated with physical modifications [1,2,4]. The typical examples of sample preparation include processes such as dissolving samples in a solvent, extracting analytes from a matrix, separating interfering components of a sample from the target analytes, enriching target analytes to make their detected signal stronger, and reacting analytes with some reagent to convert them into measurable derivatives, while the typical examples of sample pretreatment include changes in physical state such as freezing or crystallizing, grinding of a sample, or polishing or sputtering of the surface of a sample [1,2,5,6]. Due to the ever-growing number and constant improvement of sample preparation techniques and the technology supplementing them, such as automation and nanomaterials, many reviews and book chapters have been written both describing and classifying techniques for extractions, separations, derivatizations, enrichments, and labeling [1,2,3,4,6,7,8,9,10,11,12]. Since many analytical methods and workflow processes are multilayered or sequential, adequately developed sample preparation is not only essential for obtaining a clean sample for analysis but also for ensuring that the subsequent steps and instrumentation used in an analytical process are not negatively impacted [1,2,3,4,5,6]. Because of this, the sample preparation/pretreatment steps of a given method greatly impact the costs, time, and overall success of an analytical process [1,2,3,4,5,6,13]. More specifically, sample preparation is estimated to account for approximately 66–80% of sample analysis time, introduce much of the error in interlaboratory analyses, hinder the identification of sources of error arising from multiple difficulties, introduce environmental hazards due to the large volume of hazardous solvents and waste generated, and present health hazards to technicians or operators involved in a process due to exposure to large volumes of harmful solvents and residues involved in processes such as extraction [3,4,10]. In brief, sample preparation is often the linchpin of an analytical process or protocol since it is central to their validity, utility, and feasibility, which can ultimately determine the method chosen for approaching a problem. Because of the large number of analytical techniques in use, there is no universal method of sample preparation or pretreatment. The ideal method of sample preparation will need to be tailored to the process being used and is dependent on the nature of the target analytes, the matrix, and any separation steps that will need to be applied before the final analysis [3,4]. Classically, most sample preparation processes utilize solvent-based extraction techniques such as liquid–liquid extraction (LLE), solid–liquid extraction (SLE) or Soxhlet extraction that utilize large quantities of organic solvents that are immiscible with water to separate out target analytes, which is unfavorable from both an environmental and operator safety point of view as well as an analytical point of view due to their time requirements, loss of analytes, and multistep procedures [1,2,3,4,5]. In response to this, techniques utilizing smaller amounts of solvents such as solid–phase extraction (SPE), pressurized liquid extraction (PLE), microwave-assisted extraction (MAE), and many others have been developed as faster, cheaper, and simpler alternatives for extraction and separation from solid and liquid samples in addition to being easily integrated with automation, high-throughput setups, and miniaturization [1,2,3,4,5,6,9,10,11,14,15]. In this paper, we have reviewed the literature and report on several of the most prominent sample preparation approaches used with medical diagnostics developed in roughly the past decade (2010–2020). This includes methods of extraction and separation for use in point-of-care (POC) or clinical laboratory settings, high-throughput methods for use in centralized laboratories, portable devices that combine sample preparation and detection in one unit such as biosensors or microfluidic devices, and novel methodologies using established techniques like mass spectrometry (MS). As an additional note, at the time of writing this review, a large number of novel diagnostic methodologies are being developed in response to the coronavirus disease 2019 (COVID-19) pandemic, and, as such, some methods for diagnosing infectious diseases related to COVID-19 may have been overlooked.

## 2. Medical Diagnosis

### 2.1. Diagnostic Methods and Their Importance

**Figure 1:**
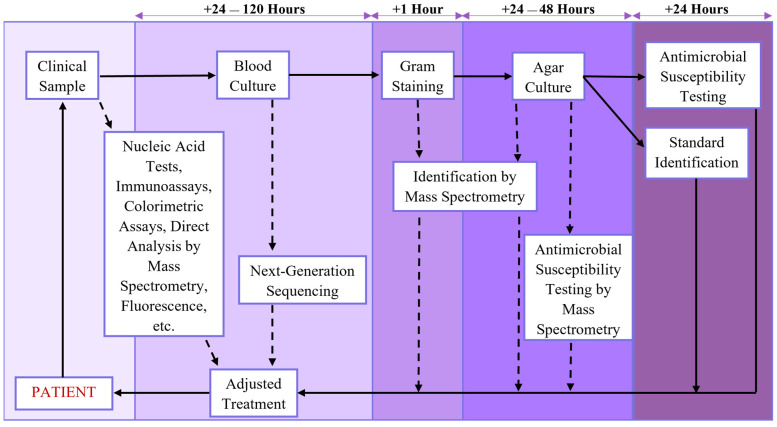
An example diagnostic workflow showing a standard blood culture procedure (solid lines) and various intervention points and techniques for reducing time (dashed lines).

Of the many analytical processes in which trends such as these have been observed, some of the most important are in medical diagnosis. In addition to the usual hurdles that sample preparation presents in analytical processes, the process of diagnostic testing in particular often requires other factors to be taken into consideration, such as the effects of collecting analytical samples on a patient, the clinical utility of the testing methodology being chosen, the cost of testing to the patient, and whether the same testing procedure will need to be repeated in the future [16,17,18,19]. Additionally, in the case of infectious diseases, diagnostic methods with adequate specificity and detail are important for preventing established pathogens from acquiring antimicrobial resistance due to prescribing broad-spectrum rather than targeted antimicrobial drugs, identifying newly emerging and reemerging infectious diseases, and properly monitoring for outbreaks of infectious diseases [19,20,21]. Infectious diseases, in particular, tend to have these difficulties due to the nature of the agents that cause them, their transmissibility compared with chronic and lifestyle-associated diseases, and the broad range of methods of diagnosing the diseases caused by them that have been developed, which can range from clinical diagnosis based on signs and symptoms being exhibited to molecular methods of laboratory diagnosis that identify the exact strain of the pathogen [20,22,23,24]. As shown in Figure 1, the routine “gold standard” methods used for diagnosing patients with an infectious disease can require several time-consuming steps due to the time needed to culture and characterize pathogens from patient samples [24,25,26]. This has led to the increased use and development of diagnostic methods to streamline the process and improve patient outcomes by decreasing the time needed to identify the cause of an infection, determine whether it is a resistant strain, and adjust patient treatment [27,28,29]. This vast range of techniques and their utility in identifying not only infectious diseases but also non-transmissible conditions can be attributed to the progress in technologies that support precision medicine over the last decade, including advances in microfluidic devices [30,31,32,33,34], next-generation sequencing (NGS) and nucleic acid amplification (NAA) methods [35,36,37,38], mass spectrometry (MS) techniques [29,38,39,40], laboratory automation [41], power sources for medical devices [42], smart materials and nanomaterials for imaging and sensing [8,43,44,45], biosensing technologies [34,43,46,47,48,49,50], smart devices for providing mobile power sources and computing power [50,51,52,53], data analysis techniques such as machine learning (ML) [54,55,56,57], and improved modeling of disease spread [58].

### 2.2. Obstacles and Considerations for Diagnostic Methods

Despite these leaps and bounds in medical technology and diagnostic methodologies, many barriers to standard clinical diagnosis remain, such as physician hesitance to adopt new diagnostic methodologies with small bodies of evidence, slow reimbursement from third-party payers for molecular diagnostic techniques, the need to promptly identify drug-resistant and novel pathogens, the need to diagnose culture-negative infections, the inability to promptly differentiate bacterial and viral respiratory infections, and the need for simple and easy to use testing methods when training technicians [24,26,27,59,60]. In addition to the roadblocks to standard clinical care, further limitations exist for the large variety of resource-limited settings such as low- and middle-income countries (LMICs), the Global South, rural areas, and disaster-stricken regions, including the lack of reliable infrastructure for communication, clean water, and power sources, the tendency for infectious diseases to spread rapidly due to crowding after a natural disaster, lack of access to expensive reagents and devices, lack of trained personnel for complex diagnostic techniques, and increased exposure to disease vectors like insects and livestock [25,61,62,63,64]. Biochemical methods like enzyme-linked immunosorbent assays (ELISA) and molecular diagnostic methods, particularly those utilizing nucleic acid tests (NATs) or nucleic acid amplification tests (NAATs) based on the polymerase chain reaction (PCR) and its many derivatives, have proven to be invaluable in overcoming these obstacles when diagnosing both infectious diseases and non-transmissible conditions in a clinical setting due to their relatively simple operation, quantitative results, molecular-level identification of biomarkers, high sensitivity, and relatively low cost in most cases [19,24,25,27,28,38,65,66].

### 2.3. Point-of-Care Diagnostics

**Figure 2:**
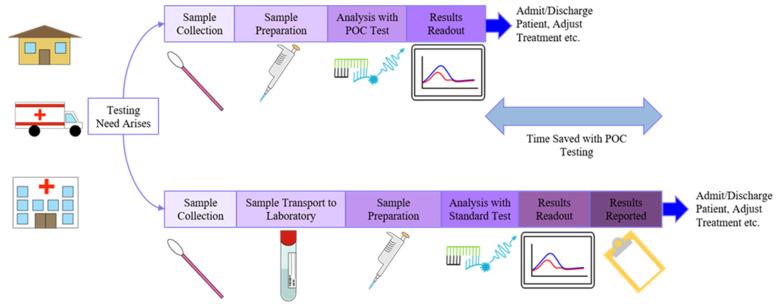
Comparison of the workflows for diagnostic methods in point-of-care (POC) settings (**top**) and standard centralized laboratory settings (**bottom**).

Beyond their advantages in traditional clinical laboratory settings, biochemical and molecular diagnostic methods have proven to be quite capable of adapting to point-of-care (POC) settings, such as a bedside in a hospital, at home, or in field conditions [67,68]. According to the World Health Organization (WHO), devices for point-of-care testing (POCT), the process of diagnostic testing at or near a patient, should meet the “ASSURED” criteria: affordable, sensitive, specific, user-friendly, rapid and robust, equipment-free, and delivered to the end user [67]. As depicted in Figure 2, POCT can save considerable time in the process of screening for disease or making decisions on patient treatment due to samples not requiring transport and results being acquired at the POC [27,60,67,68,69]. Due to the previously listed innovations in technology as well as the low cost, portability, and quick results of POCT for various health conditions has risen greatly in the past decade both as a supplement to centralized laboratory testing and as a frontline tool for diagnosis, disease surveillance, and health monitoring particularly for nations in the Global South with high disease burden or a lack of centralized laboratory testing infrastructure [67,70,71]. Additionally, because of these factors, POCT has also found uses in other applications in areas such as veterinary testing, space travel, sports medicine, emergency medicine, and ecoimmunological studies [68,69,71]. Regardless of the testing setting or method used, however, sample preparation is required. POCT in particular often faces additional challenges compared with the laboratory-scale testing methods that many methods are based on since clinical samples collected from patients at the POC are often in complex matrices such as whole blood, urine, or saliva, and any sample preparation has to be easily performed at the POC [53,67,72].

### 2.4. Sample Preparation in Diagnostics

**Figure 3:**
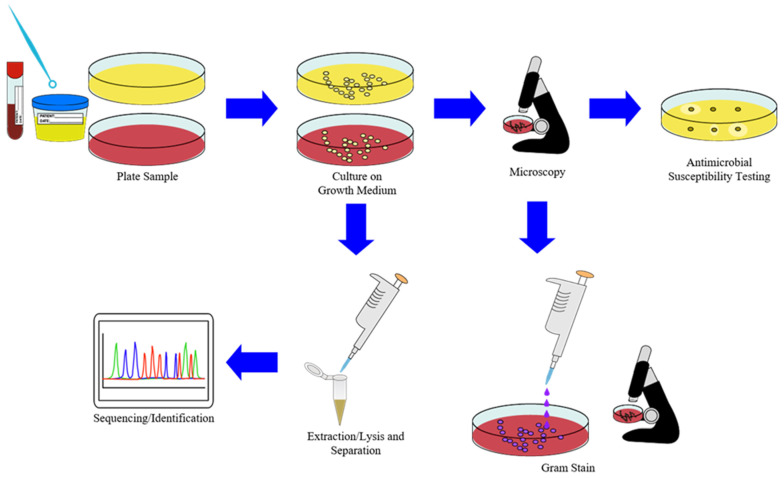
Steps in a standard microbial culture or gold standard method of identifying pathogens from clinical samples.

**Figure 4:**
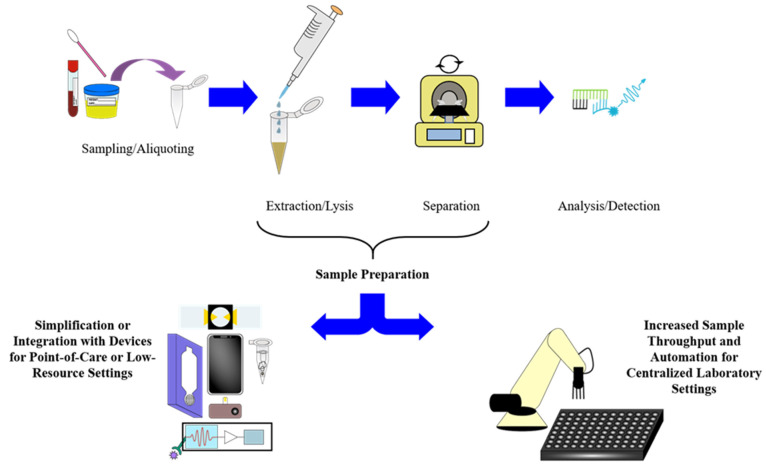
Simplified workflow of molecular diagnostic methods and two approaches to improving sample preparation in different settings.

As shown in Figure 3, the sample preparation for gold standard or culture-based methods of diagnosing infectious diseases in a traditional laboratory setting generally involves preparing growth media for pathogens, staining them with Gram’s method, extracting genetic material or other biomarkers, separating target analytes for sequencing or other identifications, and exposing of pathogens on growth media to antibiotics in order to determine antimicrobial resistance [21,24,25,26,27,28,29,40]. Since much of this sample preparation process is either impractical for use in POCT methods or has a much longer turnaround time as well as less sensitivity and specificity than biochemical or molecular diagnostic laboratory methods, many of the methods developed over the past decade have focused on either making the extraction and separation steps compatible with POC platforms or increasing the sample throughput of biochemical and molecular diagnostic methods used in clinical laboratory platforms [21,24,25,26,27,28,29,38,39,41,46,66,70]. As previously mentioned, the large number of advances in areas such as microfluidics, advanced materials, and biosensors, as well as the growing ubiquity of smartphones, has greatly supplemented this process for POC devices and methods while advances in laboratory automation, extractions and separations, and high-throughput assay platforms, such as microplates, have analogously supplemented the process for centralized laboratory methods, as shown in Figure 4 [39,40,41,42,43,44,45,46,47,48,49,50,51,52,53,67,68,69,70,71,72,73]. For molecular methods, including NAATs in particular, the greatest bottleneck in this process usually consists of extracting the target biomarkers by lysing the pathogens in a collected sample and separating or purifying the target analytes in order to proceed to amplification or detection [26,37,53,58,67]. In response to this, a great number of extraction/lysis methods have been developed for use in tandem with diagnostic methods utilizing nucleic acids as the target biomarkers, as they are both simple to apply to POC settings while maintaining sensitivity and specificity and are amenable to scaling up for high-throughput clinical laboratory testing for a large number of pathogens [21,26,74,75,76,77,78]. Similarly, many separation methodologies have been developed to complement the number of extraction methods for use with analytical setups in various settings and sizes [76,77,79,80,81,82].

## 3. Biosensors

**Figure 5:**
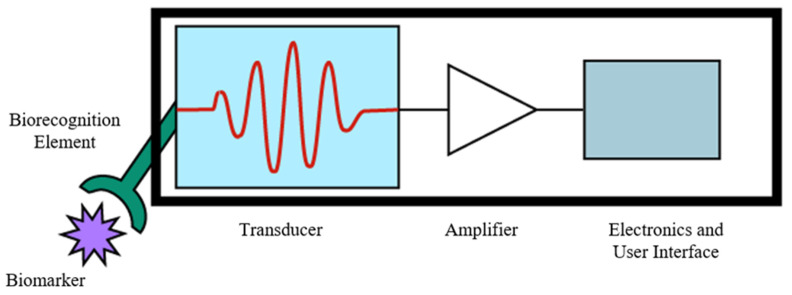
Generalized schematic for the components of a diagnostic biosensor: the target biomarker (protein, nucleic acid sequence, antibody, etc.), the biorecognition element (peptide, antibody, enzyme, etc.), the transducer (optical, electrical, plasmonic, etc.), and the electronic readout and user interface.

With the rise of personalized medicine and increasing research into technologies supporting it, biosensors are becoming an increasingly utilized technology for many diagnostic methodologies [82]. The IUPAC definition of a biosensor is “a device that uses specific biochemical reactions mediated by isolated enzymes, immunosystems, tissues, organelles or whole cells to detect chemical compounds usually by electrical, thermal, or optical signals”, or, more concisely, a device that converts a binding event between a target biomarker or pathogen and a recognition element into a measurable, quantifiable signal [79,83]. In very broad terms, a biosensing device consists of a biorecognition element that detects a target biomarker, a transducer that converts this detection into a signal, and an amplifier and electronic interface [47,48,79,83,84]. As depicted in Figure 5, each of these components is usually contained in a single, monolithic device that performs the sample preparation, testing, and readout and can be developed for detecting a wide array of biomarkers using a variety of signal transduction mechanisms, providing an incredibly useful setup for POC diagnostics due to its portability, simple operation, direct result readout, high sensitivity and specificity, and minimal sample preparation requirements for samples such as whole blood [48,83,84]. For these reasons, biosensors have gained widespread use as a method of monitoring glucose levels for patients with *diabetes mellitus*, and the market for POCT biosensors is expected to grow to $33 billion by 2027, mainly driven by molecular diagnostic devices [48].

### 3.1. Biorecognition Elements

In general, biosensors are described by either the biorecognition element utilized for detection or the signal transduction mechanism used for reading the biochemical signal [47,48,83,84]. There are many different biorecognition elements that have been developed for a wide range of targets, including enzymes, antibodies, DNA, RNA, peptides, aptamers, and even fully synthetic materials such as molecularly imprinted polymer (MIP) [34,83]. Many early biosensors, including the now-common glucose meter, utilized enzyme-based biorecognition elements due to their high selectivity, rapid turnover rate, and compatibility with multiple transduction methods [83,85]. Antibodies have become more common as biorecognition elements for reasons similar to their use in ELISA-based assays: high specificity and affinity for antigens in biological samples, the large number of antigen targets available, their ability to detect microbes, and the increasing commercial feasibility of producing engineered antibodies, recombinant antibodies (rAbs), monovalent antibodies, and single-chain variable fragments (scFvs) [79,83,84]. DNA and RNA-based biorecognition elements have grown in usage for similar reasons due to the specificity and large number of DNA and RNA probes available, as well as being easily multiplexed for screening [49,83,84]. In recent years, engineered recognition elements such as peptides, short chains of amino acids, MIPs, polymer matrices that can be implanted with arbitrary target molecules, and aptamers, short strands of oligonucleotides or peptide domains, have emerged as biorecognition elements due to their high selectivity, specificity, and affinity for their targets, as well as the ability to tailor their structure to a particular target biomarker [34,43,83,84,86,87,88].

### 3.2. Signal Transduction Methods

Several methods of signal transduction have become commonly used for biosensing devices, including optical, electronic, gravimetric, electrochemical, and electromechanical signals [43,46,48,79,83,84,85,89,90,91,92,93]. Optical signal transduction methods, which detect signal responses from the binding of biomarkers to biorecognition elements on a surface by measuring changes in refractive index, absorption, or other spectroscopic measurements, have emerged as probably the most common method used in POC diagnostic devices over the past decade [46,89,90,91,92,94]. Of the various optical signal transduction techniques, surface plasmon resonance (SPR), which senses the changes in plasmon oscillations of a surface due to the changes in adsorption, fluorescence, refractive index, or Raman scattering that result from target analytes binding with biorecognition elements on a surface and its derivatives like surface plasmon resonance imaging (SPRi) and localized surface plasmon resonance (LSPR) are probably the most common due to advances in plasmonic materials, portability, and ease of multiplexing [43,46,73,89,90,91,92,94,95]. While optical methods are the most common method of transduction in POC biosensors, other methods include electrochemical impedance spectroscopy, which measures changes in electrical impedance due to binding of target analytes [46,79,84], electromechanical methods such as quartz crystal microbalance (QCM), and atomic force microscopy force spectroscopy (AFM-FS), which measures changes in electrical signal resulting from binding between target analytes and biorecognition elements on a probe [46,83,93,95,96], and electrical methods such as biological field-effect transistors (Bio-FETs), which measure the changes in electrical signal in a semiconducting field-effect transistor that occur due to binding between target analytes and biorecognition elements [97,98].

### 3.3. Progress in Biosensors for POCT

As previously stated, many advances in biosensors have emerged over the past decade due to advances in materials, fabrication techniques, and smart devices enabling their use in POC settings [43,46,47,48,49,50,51,73,84]. Advances in microfluidics and nanomaterials, in particular, have been highly beneficial in creating total analysis systems (TAS) that integrate sample preparation and detection in one device, multiplexed detection platforms for a large number of biomarkers, and biorecognition elements for a number of infectious and chronic diseases [30,49,83,94,99]. Improvements in the detection of optical signals such as fluorescence and Raman scattering have allowed for greater sensitivity and lower limits of detection in the biosensing platforms that utilize them, and improvements in fabrication have allowed for an increasing number of signal transduction methods that can be integrated with smart devices for POCT [89,90,91,92,93,94,95,100,101]. More recently, with the rise of cheap biosensing technologies and smart devices, wearable biosensors for noninvasive and real-time health monitoring have started to become a trend in diagnostics [102,103]. Most recently, however, biosensors have shown great utility in the COVID-19 pandemic for POCT and the development of novel diagnostic assays [104].

## 4. Integrated and Portable Sample Preparation Devices

While biosensors show great promise as diagnostic tools, they are currently limited to mostly clinical settings and non-transmissible diseases due to their specificity to certain biomarkers, incompatibility with certain complex samples, and relatively high cost and turnaround time when compared to biochemical or molecular techniques such as ELISA and PCR [70,83]. As a result of this, biochemical and molecular assays remain the gold standard methods of diagnosis for certain infectious diseases and one of the primary focuses in POCT [32,70,77,105,106]. Consequently, much research over the past decade has been devoted to streamlining and improving the sample preparation required for these methods so that it can be performed more easily in POC and low-resource settings (LRS) for lower costs and with adequate analytical sensitivity and short turnaround time [105,106,107]. Within this research area, several trends have been observed, including chip or cartridge systems with integrated sample preparation [32,105,106,107,108,109,110] and portable or simplified systems for extractions and separations used with biochemical or molecular diagnostic techniques [111,112,113].

### 4.1. Integrated Sample Preparation Systems 

**Figure 6:**
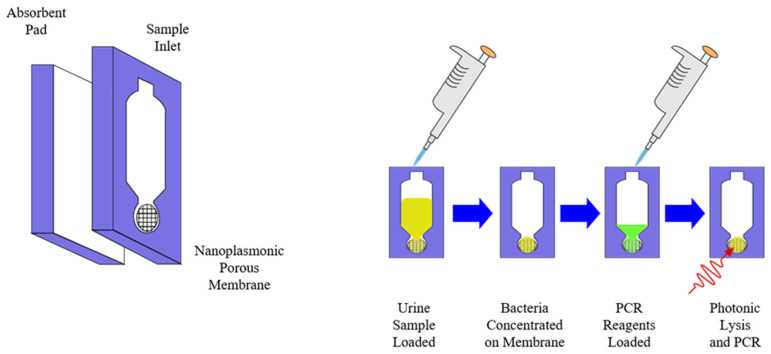
Cartridge-based diagnostic assay for urinary tract infections (UTIs) using a membrane and absorbent pad to separate pathogens from urine and then performing photonic lysis and polymerase chain reaction (PCR) in one step using a portable LED source. From Cho et al., 2019 [107].

**Figure 7:**
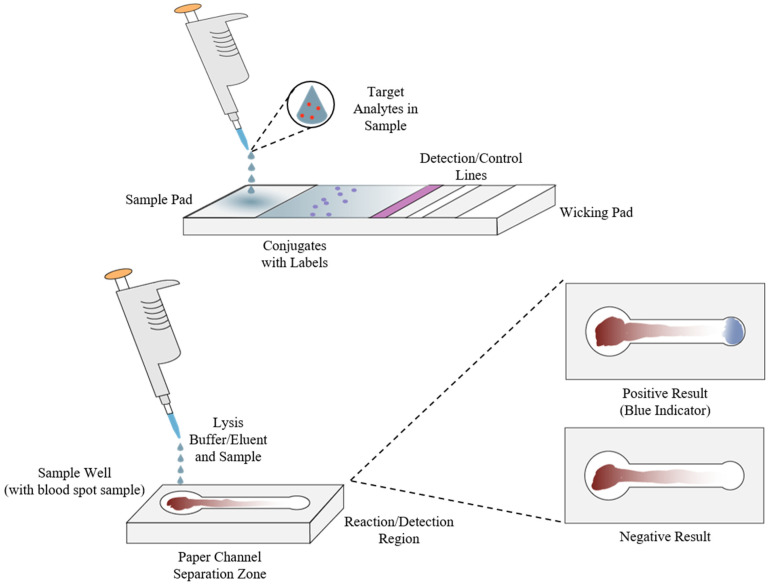
Generalized lateral flow assay (LFA) or dipstick test (**top**) and microfluidic paper-based analytical devices (μPADs) used for diagnosis (**bottom**).

As previously stated, immunoassays and nucleic acid tests or nucleic acid amplification tests (NATs or NAATs) are some of the most widely used biochemical and molecular diagnostic methods, particularly ELISA and PCR [67,77]. One of the main limitations to their use is the need for simple and quick extraction and separation of analytical targets from clinical samples and the need for complex laboratory equipment such as a thermocycler in order to carry out the amplification process [26,37,67,74]. To overcome this, one approach has been to design systems with integrated sample preparation and detection, commonly called “molecular cartridge-based” or “chip-based” tests that allow for clinical samples to be analyzed with minimal equipment requirements [27,74,107,108]. As shown by the example in Figure 6, this approach often utilizes specifically designed materials for the extraction, separation, and amplification steps of the NAAT sample preparation process and portable equipment such as LED lasers and smartphones for the detection and quantification steps [107]. Just as with the other methods mentioned, advancements in nanomaterials, microfluidics, and portable power sources have greatly benefited this area in the past decade with advanced materials making techniques such as photonic lysis of samples and ultrafast amplification of nucleic acids possible, paper-based microfluidics allowing for cheaply manufactured and reliable separation of analytes from complex clinical samples like urine or whole blood, and more portable power sources making devices for POCT in resource-limited settings as well as online air monitoring possible [33,105,107,108,109,110,111,112,113,114,115,116,117,118,119]. Similarly, Figure 7 shows examples of both a common lateral flow assay (LFA) or diagnostic test strip, such as those found in home pregnancy tests, and a microfluidic paper-based analytical device (μPAD), which have emerged as a cost-effective, rapid, multiplexable, biodegradable, sensitive, and specific biochemical diagnostic method compared to traditional lateral flow immunoassays and polydimethylsiloxane (PDMS) microfluidic setups [31,32,33,34,82,106,109,110,118]. Paper, in particular, has emerged as an attractive medium for this application due to its cheapness and utility as a manufacturing material, compatibility with small volumes of fluids found in clinical samples, and liquid transport properties [119]. Over the last decade, μPADs and similar immunoassays have shown great promise as diagnostic methodologies in POC and low-resource settings due to the variety of production methods that can be employed in manufacturing them, as well as the wide array of biomarkers that can be utilized for detection, similar to biosensors [31,119,120]. Applications have also been found in high-throughput drug screening and environmental monitoring [120,121], colorimetric measurement of proteins in urine [122], and even plasmonically enhanced immunoassays utilizing synthetic polymers in place of cellulose [123].

### 4.2. Standalone Sample Preparation Systems

**Figure 8:**
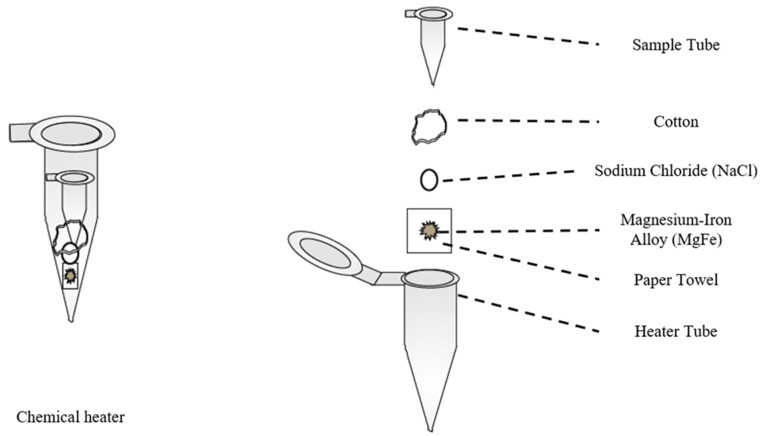
Portable chemical heater (**left**) for rapid lysis in point-of-care (POC) or low-resource settings (LRSs). Exploded view of the components and assembly (**right**). From Buser et al., 2016 [112].

**Figure 9:**
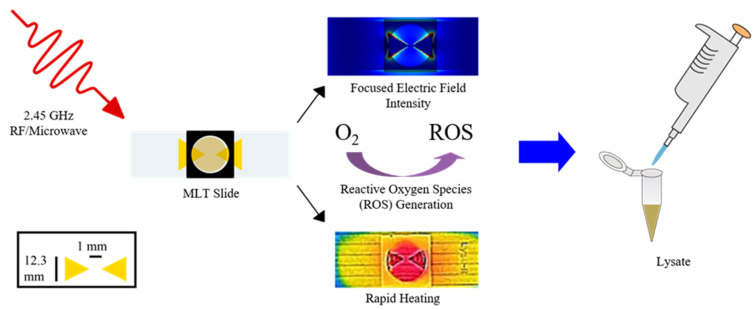
Lyse-It^®^ devices for microwave-assisted extraction and lysis. The rapid heating, focused electric field intensity, and reactive oxygen species (ROS) responsible for lysis arise from the incident microwave photons. From Geddes et al. [129,130,131,132,133,134,135,136].

**Figure 10:**
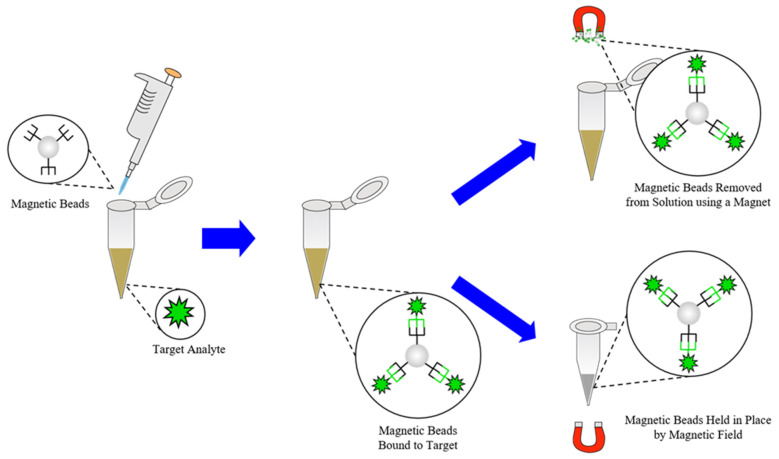
Magnetic bead-based extraction used for rapid separation or magnetic pelleting of target analytes. Paramagnetic beads can either be used to separate target analytes from an extracted sample (**top right**) or hold them in a sample container while the sample matrix is removed (**bottom right**) using only an external magnetic field.

Just as many cartridge and chip systems that integrate sample preparation and detection into one device have been developed, many standalone systems for sample preparation steps such as extraction and separations have as well. As mentioned previously, the extraction of target analytes such as nucleic acids or antibodies from clinical samples is often the primary goal of any extraction steps involved in sample preparation [9,10,14,26,37,53,57,67,77,80,124]. To accomplish this, several different approaches to extraction/lysis of pathogens have been developed, including chemical, enzymatic, mechanical, sonication, thermal, electrical, and focused radiation methods [74,76,125,126]. One notable example that has been designed for use in POC or LRSs can be seen in Figure 8, wherein Buser et al. created a portable chemical heater designed to lyse *Staphylococcus aureus* and human respiratory syncytial virus (RSV) in nasal samples via enzymatic lysis with achromopeptidase (ACP) followed by thermal deactivation of the enzyme with a chemical heater [112]. The utility of this method comes from the low costs and minimal requirements to lyse pathogens in less than 5 min since ACP can be readily purchased and stored while the components of the chemical heater can be produced from common laboratory supplies [112]. In a similar vein, Shetty et al. have created a single tube sample preparation method that can lyse *Mycobacterium tuberculosis* bacteria, disinfect the sample, and amplify the target DNA in a single, 60-min step using a heating block and amplification reagent mix [127]. Lee et al. have applied similar approaches to create a portable, low-cost lysis apparatus that utilizes a piezoelectric diaphragm and either glass beads or sodium dodecyl sulfate (SDS) to lyse *Bacillus subtilus* bacteria in approximately 30 s [113], as well as a lysis cartridge for in situ monitoring of waterborne bacteria using corona discharge to generate reactive oxygen and nitrogen species such as ozone for lysis [128]. Geddes et al. have taken a similar approach by developing a lysis setup of planar metal structures on a glass substrate called microwave lysing triangles (MLTs) or Lyse-It^®^ devices that utilize a common radio frequency (RF)/microwave source (300 MHz–300 GHz), microwave ovens, to prepare clinical samples for downstream detection in 30–60 s, as depicted in Figure 9 [125,129,130,131,132]. The utility of this approach comes from several of the factors needed for POC sample preparation methods such as simple operation, minimal equipment requirements, and lack of cold chain requirements for samples treated with MLTs, as well as the ability to fragment common target biomarkers such as nucleic acids, enzymes, and proteins in a tunable manner for downstream analysis [133,134,135,136]. While similar extraction techniques, termed microwave-assisted extraction (MAE), have found use with environmental, geological, and biological samples [137,138,139], some researchers such as Ahirwar et al. have reported using microwave irradiation to reduce the time needed for diagnostic ELISAs from 2 h to less than 5 min via non-thermal effects [140]. Besides lysis-focused extraction techniques, several other off-line techniques have been developed to simplify and reduce the turnaround time required for the extraction or enrichment steps of sample preparation. While integrated systems have been shown to approach the separation of target analytes from lysate by using microfluidic setups or silica-based stationary phases for separating target analytes from samples [80,117,141], in recent years, several standalone systems have begun utilizing magnetic nanoparticles (MNPs) or magnetic beads for separating target analytes from lysate of clinical samples or boosting their signal to noise ratio (SNR) for detection [45,81,142,143]. An example of their use as a separation technique can be seen in Figure 10, in which they are used to separate some biomarker targets from some lysate. As demonstrated by Mulberry et al., magnetic nanoparticles have great utility as a separation method in POC and LRSs since they can be easily stored, tailored to bind with a specific biomarker, do not require pipettes or lab equipment, and have a high amount of analyte recovery compared to normal methods such as silica filter-based methods [81]. In terms of enrichment or signal enhancement, Neely et al. have demonstrated the ability to detect Candida yeasts in whole blood specimens using MNPs as capture probes for target DNA sequences and as magnetic probes for T2-magnetic resonance detection [143]. While this approach may not sound ideal for diagnosis in LRSs, it does represent a diagnostic method with higher sensitivity and faster turnaround time (≤3 h) compared to the established PCR methods for *Candida* (≤12 h) and standard blood culture methods (≤2–5 days); in addition, POC nuclear magnetic resonance (NMR) spectrometers and magnetometers are an emerging trend in diagnostics [45,143].

## 5. High-Throughput Diagnostic Methods in Laboratory Settings

While many advances have been made in sample preparation for diagnostic methods in POC and LRS, centralized laboratories in clinical or hospital settings can provide greater accuracy and precision in testing and benefit from improvements to sample preparation methodologies [16,39]. To allow for greater sample throughput and simultaneous preparation and analysis, microplates are commonly used for high-throughput assays, and clinical laboratory automation is becoming increasingly common [41,144,145]. As advancements in various omics sciences and precision medicine for diagnosis and the techniques that they utilize, such as MS and NGS, continue to be made, the need for greater sample throughput, faster turnaround times, and reliable sample preparation grows as well [39,41,75]. Over the last decade, several trends in sample preparation have emerged in response to this growing need, complemented by the previously described advances in MS and NGS sequencing techniques in diagnosis and antimicrobial stewardship [27,29,35,36,37,38,39,40].

### Modified Microplates and Laboratory Automation for Diagnostic Sample Preparation

**Figure 11:**
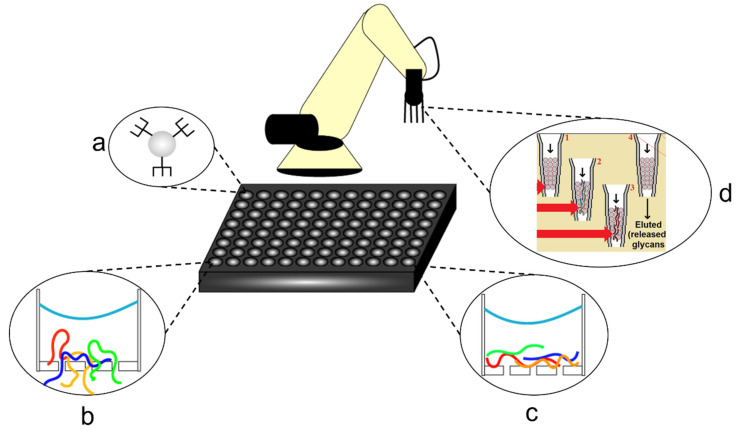
Microplate-based methods for high-throughput and automated sample preparation: (**a**) magnetic beads used for high-throughput separations or magnetic pelleting; (**b**) MStern blot adsorption of proteins to the well for separation; (**c**) filter-aided sample processing (FASP) for separating proteins using molecular weight cutoff (MWCO); (**d**) in-tip digestion of protein samples using an automated, modified pipette and a 96-well plate robot. From Mafra et al. [147], Berger et al. [152], Switzar et al., Yu et al., Wisniewski et al. [153,154,155], and Yang et al. [41,156].

Microtiter or microplate-based assays have been in use since the 1960s, and their dimensions and well densities were standardized in 2003 with either 96, 384, 1536, or 3456-wells per plate [144]. Since then, many high-throughput methods of screening and diagnostic assays have been developed, utilizing them to increase sample throughput, reduce statistical error, and more recently utilize their standard sizing to enable continuous, automated sample preparation and handling [41,144,146,147,148]. Advances in mass spectrometry and its applications to areas such as clinical proteomics, as well as the rise of next-generation sequencing to pathogen metagenomics and antimicrobial stewardship, gave rise to the concept of integrating sample preparation methods with microplates and automation equipment such as pipetting arms [41,147,148,149,150,151]. As shown in Figure 11, many sample preparation methods that have been integrated into microplate platforms are centered around clinical proteomic techniques and, therefore, focus on techniques such as lysis, digestion, separation by molecular weight, or denaturation [147,152,153,154,155]. As described by Mafra et al., a high-throughput, 96-well microplate version of parallel single-droplet microextraction (Pa-SDME) was designed for use with magnetic ionic liquids for analyte separation and was able to achieve complete and consistent extraction within 90 min in addition to being fully automated [147]. Similarly, Berger et al. developed a high-throughput filter technique for use with 96-well microplates in clinical proteomics named MStern blotting, which is capable of processing 96 urine samples in a day by using a polyvinylidene fluoride membrane to bind proteins in a certain molecular weight range to the inside of the wells during washing steps [152]. By comparison, Switzar et al., Yu et al., and Wisniewski et al. developed a technique termed filter-aided sample preparation (FASP) for use with 96-well microplates in clinical proteomic diagnosis [153,154,155]. The FASP method utilizes a molecular weight cutoff (MWCO) membrane to filter out digested peptides above a certain molecular weight for downstream analysis and allows for all sample preparation steps in a proteomic analysis to be carried out in a single 96-well microplate [153,154,155]. Building off of the microplate-based diagnostic assay and integrated sample preparation concepts, Solovjev et al. have developed a microplate-based, chemiluminescent NAAT assay [156], and Nichols et al. have developed a microplate designed to prepare clinical samples for detection via lysis by microwave heating [157]. As observed with POC-focused methods, smart devices have also demonstrated clinical utility as mobile microplate readers for diagnostic assays, as reported by Wang et al. [158]. Focusing more directly on automation tools for sample preparation, Yang et al. reported a method integrating an automated liquid with chemoenzymatic substrates in the pipette tips for digesting glycoproteins from urine samples used in conjunction with 96-well microplates [159]. Similarly, Mishra et al. have developed a method for automating high-throughput ELISA screenings for prostate cancer biomarkers found in whole blood using a centrifugal microfluidic system termed lab-on-a-disk (LoaD) that allows for multiplexed, multistep, multi-reagent screening protocols [160].

## 6. Closing Remarks

Over the last decade, a large number of analytical devices and methodologies have been developed for the purposes of improving sample preparation in medical diagnostics. These advancements have been made to address many of the variables that lead to negative patient outcomes in diagnostic testing in addition to current unmet needs in diagnosis and growing concerns such as antimicrobial stewardship, emerging infectious diseases, and testing needs in low-resource settings. As progress in other research areas like materials science, synthetic biology, robotics, energy storage, microfluidics, next-generation sequencing, consumer electronics, and data science has continued, it has found numerous applications in diagnostic methodologies and led to further innovation in sample preparation techniques. These have manifested in several distinct research trends in diagnostic methodology and sample preparation that we have reviewed here: biosensor systems, portable systems with integrated sample preparation for point-of-care testing, standalone systems for point-of-care and low-resource settings, microplate-based high-throughput methods, and automated methods for centralized laboratories. Over the next decade, it is likely that these trends will continue with the rise of personalized medicine, molecular diagnostics, and the effects of emerging infectious diseases such as the COVID-19 pandemic. These trends are also likely to be supplemented by other growing fields such as biomedical engineering and machine learning, whose applications have become apparent in recent years. Overall, the field of diagnostic methodologies and with it, sample preparation, has not yet reached its full potential.

## Data Availability

Not applicable.
